# Psychometric Validation and Factor Structure of the Minnesota Satisfaction Questionnaire—Short Form in the Romanian Private Healthcare Context

**DOI:** 10.3390/healthcare13233132

**Published:** 2025-12-01

**Authors:** Bogdan C. Pana, Alin Maerean, Sergiu Ioachim Chirila, Ciprian Paul Radu, Dana Galieta Mincă, Vlad Ciufu, Adrian Mociu, Nicolae Ciufu

**Affiliations:** 1 Department of Public Health, University of Medicine and Pharmacy Carol Davila, 050463 Bucharest, Romania; 2 Ovidius Clinical Hospital, 905900 Ovidiu, Romania; 3 Faculty of Medicine, Ovidius University of Constanta, 900470 Constanta, Romania; 4 Brighton School of Medicine, Brighton BN1 9PX, UK; 5 Department of General Surgery, Faculty of Medicine, Ovidius University of Constanta, 900470 Constanta, Romania

**Keywords:** job satisfaction, hospital worker satisfaction, MSQ—short form, MSQ validation, romanian healthcare

## Abstract

**Background:** The Minnesota Satisfaction Questionnaire is a widely recognized and used self-reporting instrument designed to measure a person’s satisfaction with various aspects of their job, as well as to provide comparative values regarding general satisfaction and its components. **Objective:** This study first aimed to test and validate the psychometric properties of the Minnesota Satisfaction Questionnaire–Short Form (MSQ-SF). Its second objective was to assess the job satisfaction levels of employees working within the organization and the factors influencing job satisfaction. **Methods:** This descriptive cross-sectional study analyzed the responses of 435 hospital staff members using the Romanian version of the MSQ-20 scale. **Results:** Exploratory Factor Analysis identified a three-factor structure: Task Enrichment, Autonomy Satisfaction, and Supervisory Relationships. The three-factor model with eight MSQ items discarded provided an excellent statistical fit. The MSQ-SF with a 20-item questionnaire has excellent Internal Consistency, with a Cronbach alpha of 0.935, 95% CI (0.926–0.944). **Conclusions:** The Romanian version of the MSQ-20 has excellent construct validity and consistency, and it provides reliable and comparable data on the health of the workforce.

## 1. Introduction

People’s health needs and preferences are at the core of a health system evaluation framework, and both are important objectives of health systems [1]. The relationship between the professional satisfaction of workers within the healthcare field significantly influences the quality of the services provided. Relevant studies in the field support the idea that satisfied healthcare professionals are more likely to provide higher-quality, safer, and more compassionate healthcare to patients [2]. Thus, evaluating the level of work satisfaction in different healthcare settings might provide relevant information for the management of and support for relevant measures for improving the provided services.

Varied instruments have been described, ranging from standardized questionnaires to qualitative methods. Common instruments used for measuring job satisfaction include the “Job Satisfaction Questionnaire (JSS),” which assesses satisfaction across nine dimensions, such as pay, promotion, supervision, and nature of work [3]; the “Minnesota Job Satisfaction Questionnaire (MSQ),” which is available in both long and short forms and examines intrinsic and extrinsic aspects of a job [4]; the “Job Descriptive Index (JDI),” which assesses employee attitudes towards various aspects of their work, including the work itself, pay, promotion opportunities, supervision, and coworkers [5]; and the “Employee Net Promoter Score (eNPS)”, a metric used to gauge employee loyalty and engagement by measuring how likely they are to recommend their company as a place to work [6].

One widely used tool for assessing job satisfaction with broad international recognition is the MSQ [7,8,9,10,11]. The instrument is a self-report questionnaire that measures a person’s satisfaction with different aspects of their job, developed by Weiss, Dawis, England, and Lofquist in the 1960s [4], while the core constructs have been constantly adapted, it’s measures remain relevant. It exists in both long-form (100 items, assessing 20 facets of satisfaction) and short-form (20 items, providing scores for intrinsic, extrinsic, and general satisfaction) versions. The short-form version (MSQ-SF) is structured to reflect two primary satisfaction dimensions: intrinsic satisfaction, which refers to the satisfaction derived from the work itself, such as a feeling of achievement, using one’s abilities, and having variety, and extrinsic satisfaction, which relates to external job rewards and context, such as compensation, working conditions, and supervision [4]. The MSQ is based on the work/job adaptation theory pioneered by Dawis and Lofquist [12], which posits that job satisfaction stems from the congruence between an individual’s needs and the reinforcing factors provided by their work environment, making it a valuable tool for vocational counseling, organizational research, and evaluating the effectiveness of workplace interventions. The MSQ is useful when rating job satisfaction with various aspects of a job, such as compensation, supervision, coworkers, and opportunities for advancement or achievement, on a five-response Likert-type scale.

Existing data suggest that the MSQ-SF is a valid tool for evaluating the work satisfaction of employees within healthcare units [9,10,11,13]. Simultaneously, as a self-administered psychometric tool, the instrument should be translated and adapted to the local context in which it is applied. The MSQ-SF was specifically selected for this study due to its widely recognized international validity, its conciseness and the speed and ease with which respondents can complete it. While other instruments, such as the JSS, are available and free, they often contain a significantly higher number of items, which can negatively impact response rates in busy clinical settings.

Validation studies of the MSQ-SF across different countries and professional groups have frequently challenged the original two-factor (intrinsic/extrinsic) structure, leading to various factor solutions. A summary of factor analytic findings across different validation studies is presented in Table 1. This variation suggests that the original two-factor model may not adequately capture the nuances of job satisfaction across diverse cultural or professional settings. Therefore, the main objective of this study was to translate the MSQ-SF and measure the instrument’s psychometric characteristics. The second objective was to evaluate the factors that influence job satisfaction among personnel working at the private hospital where the study was conducted.

## 2. Materials and Methods

### 2.1. Translation Process

To translate and adapt the MSQ-SF into Romanian, a meticulous, multi-step process was undertaken. An expert panel consisting of two highly experienced English-to-Romanian translators (one with significant expertise in medical terminology) and several researchers from the medical and management fields was established [14].

Two panel members independently performed the initial translation. The translated versions were then reviewed and synthesized to create an intermediary Romanian draft. This draft was subsequently back-translated into English by two other panelists who were not involved in the initial translation and were unfamiliar with the original MSQ-SF. The back-translated versions were then critically analyzed and compared to the original English version to ensure accuracy and address any discrepancies. Throughout this process, wording was carefully adapted to align with Romanian semantic and syntactic nuances to ensure clarity and cultural relevance.

After translation and adaptation, the draft was pilot-tested by 15 hospital department chiefs. Their feedback on its clarity and comprehension led us to make final revisions, ensuring that the items were easy to understand and respond to. Appendix A presents the Romanian version of the MSQ-SF.

### 2.2. Study Population

This study was conducted between June and July 2025 in the southeastern region of the country at the largest private hospital in Romania. This medical institution offers all medical services for adults—outpatient, inpatient, medical, and surgical services—serving a population of almost one million people. The hospital has 521 employees (full-time and part-time employees).

The survey was distributed to all 521 employees at the institution, and 435 questionnaires were completed. Some employees were on holiday or refused to participate in this study. Written consent was obtained from participants, and all necessary measures to ensure anonymity were taken. This study was approved by the ethics committee.

Although the study sample was nonrandomized, a minimum sample size of 249 respondents was initially calculated to ensure that our findings would be representative of the hospital population with a 95% confidence level [15]. This calculation was based on standard statistical practices, assuming the highest variability in the population to guarantee sufficient representation. Ultimately, 435 of 521 employees (an 83.49% response rate) were included in this study. This approach maximized the representation of hospital staff and provided a more comprehensive and robust dataset [16].

### 2.3. Data Collection

To ensure cybersecurity and confidentiality, the questionnaire was self-administered on paper in the first week of June 2025. The Research Department put a person in charge, who was previously trained by the researchers, to offer support during the survey process to anyone in need of it.

After completion, the questionnaires were deposited by the participants in a specially arranged box to preserve their confidentiality and anonymity. After the papers were collected, they were added to an electronic system by a group of employees through Google Forms and delivered to an external team of researchers for data analysis to double-check for data quality issues and possible errors. All discrepancies were reviewed and corrected.

### 2.4. Minnesota Satisfaction Questionnaire

The MSQ-SF (20 items) was the primary measure of job satisfaction. As detailed in the Introduction, this self-report instrument is based on the work/job adaptation theory and provides scores for intrinsic, extrinsic, and overall satisfaction. Respondents rate their satisfaction with various job enhancers on a 5-point Likert-type scale [17,18]. The General Satisfaction Level (objective measure) is calculated by averaging the 20 items of the original MSQ-SF.

Other variables collected included demographic traits (age, sex, type of education) and specific organizational roles and experiences (department, professional background, employment status (full time or part time), working hours, total time of activity in the workforce and time of activity within their current organization) [19].

### 2.5. Statistical Analysis

The psychometric properties of the MSQ-SF-RO were evaluated using a series of factor and network analyses.

The primary psychometric and factor analyses were conducted using JASP 0.19.3 (Amsterdam, The Netherlands) statistical software due to its transparent, open-source nature and robustness for advanced psychometric methods. However, IBM SPSS Version 25 (Armonk, NY, USA) was only employed for managing missing data, specifically for conducting the MCAR test and the subsequent imputation of mean values.

Confirmatory Factor Analysis (CFA) was the primary method used to test whether the bi-dimensional structure (intrinsic and extrinsic satisfaction) of the original MSQ-SF, theorized in the literature and confirmed in other validation studies, fits well with the data collected in the Romanian sample [20].

Exploratory Factor Analysis (EFA) was used to examine the underlying factor structure of the 20 items of the MSQ-SF in case the initial CFA did not indicate an adequate fit for the original MSQ-SF or to confirm the validity of the new structure in the Romanian context [21].

Convergent Validity was evaluated using the correlation between the identified factors of the Romanian MSQ-SF and the general job satisfaction item. The Average Variance Extracted (AVE) and Composite Reliability were calculated to assess convergence at the construct level [22].

Discriminant Validity assessed whether the MSQ-SF factors were sufficiently distinct from one another. It examined whether the square root of the AVE for each factor was greater than the correlations between the specific analyzed factor and other factors [18].

Reliability (Internal Consistency) was assessed by calculating the Cronbach alpha coefficient for the overall scale of the MSQ-SF, as well as for each subscale comprising the factors obtained through the construct validity process [23].

Network Analysis was performed to visualize the interconnections and relationships among the MSQ-SF’s validated items, providing a graphical representation of the instrument’s structure within our study [24].

Statistical significance was set at *p* < 0.05. Google Forms, IBM SPSS, and JASP software were used for data gathering and statistical analysis.

## 3. Results

Of the 521 employees of the institution, 435 completed the questionnaire, representing an 83.49% response rate.

The study population was predominantly female (362 respondents, 83.21%), compared to males (58 respondents, 13.33%) and those with an undeclared gender (15 respondents; 3.44%). The age of respondents varied from 19 to 68 years, with a mean value of 40 (±11.9 std. dev.) and a mode of 24. On average, the respondents had been employed in the institution for 3 years and 7 months (43 months), with individual employment lengths ranging from 1 month to 14 years (168 months), with a mode of 3 years and 4 months (32 months). The Shapiro–Wilk Test values for both age and institutional tenure suggested a non-normal distribution of the values (*p* < 0.001)

Regarding the professional backgrounds of the employees, healthcare professionals constituted the majority (68.0%), followed by individuals with administrative and economic (9.9%), technical (6.2%), and customer service backgrounds (4.6%). A small proportion of the sample comprised military personnel (0.7%) and undergraduates (3.9%). Additionally, 6.7% of the respondents did not declare their professional background.

When considering the activity domain within the organization, we found that most of the respondents worked in daycare departments (31.3%, representing ambulatories/outpatients, pharmacy and radiology), followed by surgical fields (20.9%, representing anesthesiology, orthopedics, and other surgical specialties), clinical fields (19.8%, representing cardiology, oncology, radiotherapy, etc.), customer service (17.5%, representing call-center, reception and admissions office), and technical and administrative fields (9.4%). Additionally, 1.1% of participants did not declare their field of work at the institution. Only 5.97% of participants occupied a senior role at the hospital, and 84.82% were full-time employees. More demographic and professional characteristics of the study population can be found in Table 2.

Among the 435 answered questionnaires obtained within this study, 88 missing item-wise responses were identified across the MSQ-SF items, representing 1.01% of the total item data (435 responders × 20 responses/ responder = 8700 total responses, of which 88 were missing). Little’s Missing Completely at Random (MCAR) test was conducted using IBM SPSS software to assess the randomness of missing data [25]. The results indicated that the data were completely missing at random (*p* > 0.05), allowing the use of imputation methods. Subsequently, missing values were imputed using the mean values for each item, allowing all 435 respondents to be retained for subsequent statistical analyses.

### 3.1. Construct Validity

#### 3.1.1. Exploratory Factor Analysis

The initial CFA of the original two-factor model (intrinsic and extrinsic satisfaction) from the MSQ-SF was conducted using JASP software. The model was decisively rejected due to poor fit and highly concerning Discriminant Validity issues. The model fit indices were $\chi^{2}$ = 701.87 with df = 167 (*p* < 0.001). Factor analysis suitability was high, with a KMO value of 0.945 and Bartlett’s Test of Sphericity being statistically significant ($\chi^{2}$ = 6481.56, df = 190, *p* < 0.001). However, the factor covariances were extremely high, with the correlation between intrinsic and extrinsic satisfaction being 0.941 and the HTMT being 0.966. These values far exceed the 0.85 threshold, definitively demonstrating a lack of Discriminant Validity between the two factors.

Consequently, EFA was performed to explore alternative factor structures. The initial EFA, using parallel analysis to determine the number of factors, suggested a model with six factors. This model indicated that seven items had loadings below 0.4 and would, thus, have been removed [26,27]. Furthermore, only three factors had eigenvalues above one, and factor correlations indicated poor Discriminant Validity [28].

Due to these unfavorable results, different combinations of factors were simulated to identify the best-fitting and favorable model. A final EFA was performed, manually specifying a three-factor model, while maintaining the Oblimin rotation and Maximum Likelihood (ML) factoring method. Oblimin rotation (an oblique method) was consistently selected because the underlying dimensions of job satisfaction are theoretically expected to be highly correlated in a real-world setting, making the use of orthogonal rotation methods (which assume factor independence) inappropriate and potentially distorting. ML was preferred over methods including Principal Axis Factoring because the former method has more statistical rigor and fit indices and significance tests are available.

The three-factor model demonstrated strong suitability for factor analysis [26]. The Kaiser–Meyer–Olkin (KMO) value was 0.927 (well above the 0.6 cutoff), and individual item values ranged from 0.84 to 0.97, indicating excellent sampling adequacy. Additionally, Bartlett’s Test of Sphericity was statistically significant (*χ*^2^ = 4059.534, df = 66, *p* < 0.001), confirming that the correlation matrix was suitable for factor analysis [29].

#### 3.1.2. Confirmatory Factor Analysis

After identifying a three-factor structure, CFA was conducted on all 20 items of the MSQ-SF. This model demonstrated excellent fit to the data, as evidenced by several additional fit indices: the Comparative Fit Index (CFI), 0.986; the Tucker–Lewins Index (TLI), 0.985; the Bentler–Bonett Normed Fit Index (NFI), 0.974; the Root Mean Square Error of Approximation (RMSEA), 0.05; and the Standardized Root Mean Square Residual (SRMR), 0.04. These values met or exceeded the standard thresholds, confirming the model’s overall fit [30].

Furthermore, the model showed strong Convergent Validity, with most standardized factor loadings being robust and above the 0.7 threshold. The R-squared (R^2^) values for the items were also substantial across all three factors.

However, a critical issue was identified with Discriminant Validity between the two factors. The factor covariance between the first two factors was extremely high (0.885), being above the 0.85 cutoff, suggesting that these two latent constructs were not empirically distinct. This concern was further supported by the Heterotrait–Monotrait Ratio (HTMT) value of 0.858, which barely exceeded the 0.85 threshold, indicating a high degree of correlation between the factors [31].

To address these issues, eight items (MSQ1, MSQ7, MSQ8, MSQ10, MSQ11, MSQ12, MSQ17, and MSQ18) with low factor loadings were removed and a new CFA was performed on the remaining 12 items [30]. Table 3 provides details of the factor loadings after Oblimin rotation was applied to the three-factor model, with eight items discarded [20]. This 12-item model accounted for 70.2% of the variance, with Factor 1, Factor 2, and Factor 3 explaining 35.9%, 19.8%, and 14.5% of the variance after rotation.

The three different factors obtained are as follows:

Factor 1—Task Enrichment: This factor, comprising items MSQ13, MSQ14, MSQ15, MSQ16, MSQ19, and MSQ20, reflects an employee’s satisfaction with the work itself and the rewards derived from it. The items collectively highlight appreciation for professional rewards (MSQ13: “My pay”), professional growth (MSQ14: “The chances for advancement”), and the intrinsic satisfaction from a sense of accomplishment (MSQ20: “The feeling of accomplishment”). The inclusion of items MSQ15 and MSQ16 further emphasizes the importance of autonomy and freedom within the job itself, which we interpret as contributing to the enrichment of work tasks.

Factor 2—Autonomy Satisfaction: Composed of the items MSQ2, MSQ3, MSQ4, and MSQ9, this factor is a classic representation of intrinsic job satisfaction. It focuses on the ability to work independently (MSQ2: “The chance to work alone”), the variety of tasks performed (MSQ3: “The chance to do different things”), and the job’s personal and social significance (MSQ4: “The chance to be ‘somebody’ in the community” and MSQ9: “The chance to do things for other people”). This factor captures satisfaction derived from having control over one’s work and its impact.

Factor 3—Supervisory Relationship: This factor uniquely focuses on the hierarchical relationship between employees and their supervisors. It consists of only two items: MSQ5: “The way my boss handles his/her workers” and MSQ6: “The competence of my supervisor in making decisions.” These items directly assess satisfaction with a supervisor’s behavior and competence, highlighting the distinct importance of this relationship in the Romanian context.

For the final CFA that employed the three-factor models and discarded eight MSQ items, the fit indices demonstrated a robust solution. *χ*^2^ was 117.74 (df = 51, *p* < 0.001) and Bartlett’s Test of Sphericity was *χ*^2^ = 4059.47 (df = 66, *p* < 0.001). The additional fit measures were as follows: the CFI = 0.992, the TLI = 0.989, the NNFI= 0.989, the NFI = 0.986, the Parsimonious Normed Fit Index (PNFI) = 0.77, the Relative Fit Index (RFI) = 0.982, the Incremental Fit Index (IFI) = 0.992, and the Relative Non-Centrality Index (RNI) = 0.992. Other fit measures were represented by RMSEA = 0.055, RMSEA 90% CI lower bound = 0.042 and upper bound 0.068, RMSEA *p*-value = 0.253, SRMS 0.03, Hoerlter’s critical *n* with α at 0.05 = 491.208 and with α at 0.01 = 553.43, and the Goodness-of-Fit Index (GFI) = 0.998. The overall KMO value was 0.927, ranging from 0.84 (MSQ6) to 0.97 (MSQ14).

The R-squared values ranged from 0.519 (MSQ9) to 0.944 (MSQ5). Factor loadings varied from 0.793 (MSQ14) to 0.891 (MSQ15) for Task Enrichment, from 0.72 (MSQ9) to 0.866 (MSQ4) for Autonomy Satisfaction, and 0.865 (MSQ6) to 0.971 (MSQ5) for Supervisory Relationships. All standard estimates were improved using the new model.

**Figure 1:**
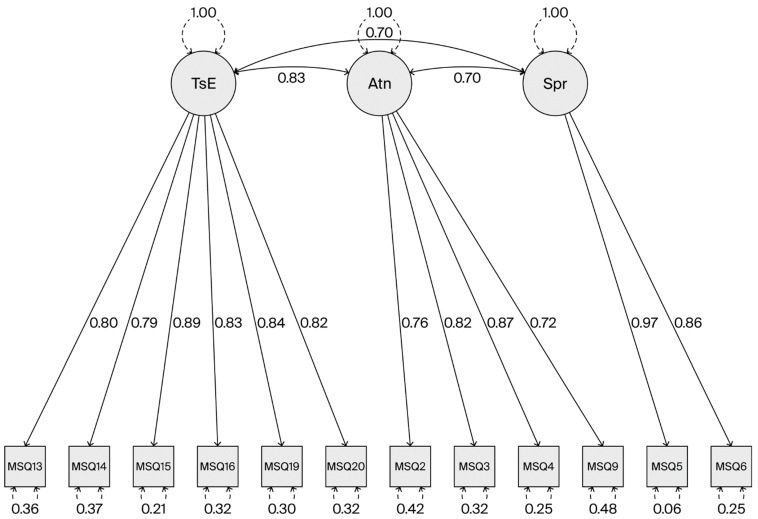
Confirmatory Factor Analysis—factor structure (TsE—Task Enrichment; Atn—Autonomy; SpR—Supervisory Relationship).

As presented in Figure 1, the covariance between Task Enrichment and Autonomy Satisfaction was 0.835 (std. error 0.022, *p* < 0.001), that between Task Enrichment and Supervisory Relationships was 0.697 (std. error 0.035, *p* < 0.001), and that between Autonomy Satisfaction and Supervisory Relationships was 0.7 (std. error 0.036, *p* < 0.001). 

The AVEs for Task Enrichment, Autonomy Satisfaction, and Supervisory Relationships were 0.688, 0.632, and 0.846, respectively. All factors showed good Convergent Validity [18].

Crucially, the Discriminant Validity was improved in this model. The HTMT values were significantly reduced, falling below the 0.85 threshold: 0.803 for Task Enrichment and Autonomy Satisfaction, 0.628 for Task Enrichment and Supervisory Relationships, and 0.654 for Autonomy Satisfaction and Supervisory Relationships; these results confirm that the three factors are empirically distinct latent constructs.

#### 3.1.3. Internal Consistency

For reliability statistics, Cronbach’s alpha coefficient was calculated to express Internal Consistency [32,33]. For the extended survey, which included the overall satisfaction item, Cronbach’s alpha was 0.94, and for the MSQ-SF with 20 items, it was 0.935. Both results reflect excellent Internal Consistency above the 0.9 cutoff [23].

In the case of the three-factor structure with eight items discarded, Cronbach’s alpha was 0.911. The three factors independently showed the following results: Task Enrichment (0.888), Autonomy Satisfaction (0.801), and Supervisory Relationship (0.824); all factors demonstrated good Internal Consistency.

#### 3.1.4. Network Analysis

**Figure 2:**
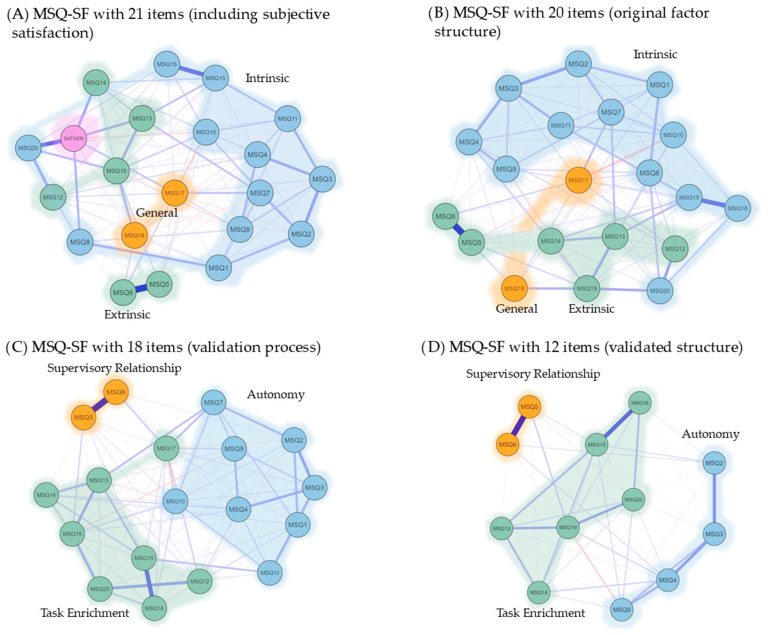
Network Plot—the underlying structure of each MSQ item correlation, considering the three latent factors obtained through the validation process.

To complement the factor analysis findings, Network Analysis was conducted to visualize the underlying structure of the MSQ-SF items. Using the EBICglasso estimator, the network plots (Figure 2) provide a graphical representation of the direct correlations between items, offering a different perspective on their relationships [34,35]. In these plots, each circle (node) represents an item, and the lines (edges) connecting them signify a direct statistical relationship. Thicker lines indicate stronger correlations, whereas clusters of colored nodes represent identified factors [24,36].

The network plots clearly illustrate the shift from the original, less-defined structures of the final validated three-factor model.

Figure 2A (21-item MSQ—SF) and Figure 2B (20-item MSQ-SF) show a highly dense and interconnected network. The numerous non-zero edges (127/210 and 122/190, respectively) and higher sparsity values (0.395 and 0.358, respectively) indicate that nearly all items are related. This high density suggests a lack of distinct and empirically separate clusters, supporting the results of our initial CFA, which found poor model fit for the original two-factor structure. The items are not clearly grouped into well-defined domains, making it difficult to interpret a clear factor structure.

Figure 2D (12-item validated structure) depicts a markedly different and clearer structure. This final model has a significantly lower number of non-zero edges (54/66) and a reduced sparsity value (0.182), visually confirming that only the most direct and meaningful relationships between the items are retained. The plot distinctly shows three separate and well-defined clusters of items corresponding precisely to the three factors identified in our final CFA: Task Enrichment, Autonomy, and Supervisory Relationships. The items within each cluster are strongly connected, whereas the connections between the clusters are minimal. This clear modularity visually reinforces the statistical findings of our CFA, providing compelling evidence of the Discriminant Validity of the three-factor model.

## 4. Discussions

### 4.1. Principal Findings and Comparisons

The MSQ-SF has demonstrated robust psychometric properties across diverse cultural contexts, establishing it as a widely recognized and continuously validated instrument for assessing job satisfaction. Recent studies, along with the existing literature, affirm its cross-cultural applicability in various nations, including Portugal [10], Poland [11], Cyprus [9], South Africa [37], Tanzania [38], Ghana [7], Kuwait [39], India [40], and Vietnam [13]. These validation efforts consistently underscore the utility of the MSQ-SF in providing reliable and valid measures of job satisfaction across different professional landscapes globally [41].

This study aimed to validate the psychometric properties of the MSQ-SF in a Romanian organizational healthcare context. Consistent with the growing body of international literature, the initial attempt to confirm the original two-factor model distinguishing between intrinsic and extrinsic satisfaction was unsuccessful. The CFA of the original model was rejected because of inadequate fit and poor Discriminant Validity, a finding that mirrors observations from validation studies conducted in countries such as Portugal [10], Cyprus [9], and Vietnam [13], where the universal applicability of the original structure has been questioned [41]. The failure of the original model may be rooted in Romania’s high Power Distance and collectivistic culture. In such contexts, the strict separation between extrinsic (organizational) and intrinsic (personal) factors may break down, as employees’ sense of self and satisfaction are deeply tied to the social and hierarchical structure of the workplace, necessitating the use of a more nuanced, multi-factor model for accurate measurement [42]. This initial finding underscores the need for culture-specific model adaptation, validating the use of an exploratory approach to uncover a factor structure more representative of the Romanian healthcare professional environment [14,23].

This study utilized a diverse sample of 435 employees in a private healthcare context, encompassing healthcare professionals (48.04%), alongside professionals working in administrative, technical, and other supportive roles. This heterogeneity is consistent with the broad application of the MSQ-SF in the international literature that extends beyond the direct clinical care provider roles typical of nursing-centric studies. This robust sample composition is crucial for confirming the instrument’s structural integrity and relevance across varied organizational functions within a single institutional setting. While often validated in clinical contexts, the instrument’s utility extends significantly beyond this sector, as evidenced by successful validation studies conducted in public service (Tanzania, *n* = 50) [38], government (Ghana, *n* = 100) [7], and finance (Bangladesh, *n* = 103) [43] fields, demonstrating its wide applicability for organizational psychology research and management practices [22].

The current sample size is comparable to those of prior studies (e.g., Vietnam, *n* = 587 [13], Portugal, *n* = 140 [10]). Its occupational mix is a key distinction: while Poland (*n* = 177) and Cyprus (*n* = 292) focused almost exclusively on nurses (92.5% in Cyprus, 100% in Poland) [9,11], this study’s inclusion of nearly 30% non-clinical administrative, technical, and customer service staff provides insights into the MSQ-SF’s limit within a complex organizational ecosystem. Demographically, gender distribution is largely consistent across most samples, with this study (83.21% female), aligning closely with results from Portugal (79.3%) and Vietnam (83.6%). However, notable differences exist regarding age and tenure. The Cypriot sample was distinctly younger (56.5% under 30), whereas the Vietnamese sample reported a significantly older mean age (μ = 54.95 years) and focused on “grassroots healthcare workers” with substantial local responsibilities [13]. The Portuguese sample (μ = 43.4 years) is closer in age profile to this study (μ = 40.81 years). Encompassing diverse age groups, educational levels and varied professional roles, it underscores the MSQ-SF’s established robustness and enhances its overall generalizability across the international healthcare landscape.

A three-factor structure with 12 items was identified as the optimal solution for the Romanian sample, consisting of Task Enrichment, Autonomy Satisfaction, and Supervisory Relationship. This finding aligns with international validation studies that also departed from the original two-factor model to find a more suitable multifactorial structure. A Vietnamese validation study, for instance, yielded a three-factor model after discarding six items: Specificity, Autonomy, and Obligation [13]. In contrast, a study conducted in Poland found a two-factor model, slightly different from the original, without discarding any items [11]. A Portuguese study by Martins et al. (2012) identified a two-factor structure after discarding 10 items consisting of Supervisor/Empowerment and Task Enrichment [10]. This variation in factor structures across different cultures, from two-factor to three-factor models, sheds light on the importance of localized validation and suggests that the underlying dimensions of job satisfaction may be perceived differently depending on the cultural and professional context [2].

Our findings bear a particularly strong resemblance to the Greek-Cypriot validation study conducted by Lakatamitou et al. (2020) [9]. While that study yielded a combined Supervisory/Autonomy factor, our analysis resulted in Supervisory Relationship as a distinct, separate construct. This factor is critical and provides a cultural explanation for model refinement. Romania’s organizational environment is profoundly influenced by its national culture, which Hofstede’s framework characterizes as exhibiting a high Power Distance (PDI) and a tendency toward collectivism, albeit one that is currently in transition [42]. The high PDI suggests a preference for centralized and hierarchical structures, where authority is rarely challenged, which can directly influence how job satisfaction is derived and reported by employees in the hierarchical healthcare setting. Furthermore, the collectivist inclination means that team cohesion, relational dynamics, and group harmony often take precedence over individual achievement, suggesting that Autonomy and Task Enrichment Satisfaction may be heavily weighted by the perceived quality of the working group and the immediate supervisor.

These cultural traits manifest in the daily operation of organizations, creating what are termed the “particularities of Romanian management” [44]. Management styles are often characterized by paternalistic leadership, a focus on personal relationships (rather than strictly formal procedures), and centralized, top-down decision-making. In the rapidly evolving private healthcare domain, where adaptability is crucial, this traditional management approach can pose challenges. However, the private sector, in particular, is simultaneously driven by global and market-based forces that demand a high degree of organizational efficiency and employee engagement. In this competitive landscape, an organization’s culture is not merely a background factor but a critical component of its competitiveness [45]. A strong, adaptive organizational culture that supports positive job satisfaction dimensions, as measured using the MSQ-SF, becomes a vital non-material resource that enables rapid response to patient demands and technological change, thereby sustaining long-term success in the market [46].

The final three-factor model demonstrated an excellent fit to the Romanian data, as evidenced by robust fit indices, including a CFI of 0.992, a TLI of 0.989, an RMSEA of 0.055 (90% CI: 0.042–0.068), and an SRMR of 0.03. These values meet or exceed the established thresholds for a strong model fit, providing statistical support for the proposed structure [47]. The conceptual rationale for these factors is well defined by the items loaded on them. The excellent CFA fit indices and the Network Analysis results provide strong counter-evidence for their Discriminant Validity. The strong connections seen in the network within these clusters, contrasted with the sparse connections between them, visually reinforce the notion that they are distinct constructs.

The EFA resulted in a three-factor model with 12 items, accounting for 70.2% of the total variance. This result is comparable to the findings of other validation studies, highlighting the robust explanatory power of the developed model [48]. For instance, the Vietnamese study’s EFA resulted in a model with 14 items that accounted for 65.766% of the variance [13]. Lakatamitou et al. (2020) produced a two-factor model with 15 items accounting for 58.0% of the variance [9]. In Martins’ study (2012), the resulting two-group model with 10 items accounted for 61.185% of the variance [10].

The Internal Consistency results from our study are highly favorable compared to other international validations [29]. The overall Cronbach alpha of 0.933 recorded for the three-factor model was higher than the figure of 0.892 reported by Walkowiak et al. (2019) for their Polish study [11] and comparable to the figure of 0.924 from a Vietnamese study [13]. The global alpha of 0.955 from the Greek-Cypriot study was slightly higher than that of our study [9]. When examining the reliability of the individual factors, our alpha values for Task Enrichment (0.888), Autonomy Satisfaction (0.801), and Supervisory Relationships (0.824) were comparable to or exceeded those found in the literature. For instance, our Task Enrichment alpha is identical to the 0.888 figure for the Greek-Cypriot study [9] and higher than the 0.87 figure for Martins’s (2012) study in Portugal [10]. Our Autonomy Satisfaction alpha is lower than the 0.873 figure for the Greek-Cypriot Supervisor/Autonomy factor [9] but higher than the 0.78 figure for Martins’ (2012) Supervisory/Empowerment factor [10]. High reliability values recorded across both the total scale and individual factors provide strong evidence for the psychometric robustness of the Romanian version of the MSQ-SF [29].

The Network Analysis conducted in this study serves as a powerful visual confirmation of the quantitative findings, aligned with the growing use of this methodology in organizational psychology and psychometrics [49,50,51]. This approach moves beyond traditional factor analysis, offering a detailed item-level perspective on construct validity [24]. For instance, a recent study on job satisfaction among radiologists used Network Analysis to visualize the intricate relationships between various work-related factors, demonstrating the capacity of this method to reveal subtle but important connections within professional contexts [49]. The findings, particularly the transition from highly dense, undifferentiated initial networks to the sparse, modular structure of the final 12-item model, are consistent with the principles of parsimony and clarity sought in psychometric Network Analysis [52]. The clear separation of the three factors visually reinforces their Discriminant Validity and confirms that they are distinct and meaningful dimensions of job satisfaction in the studied population.

The application of Network Analysis to validate the MSQ in the Romanian context is a pioneering effort, providing a methodological contribution to the field of industrial and organizational psychology within the region and for MSQ validation. By applying the EBICglasso estimator, this study confirmed the statistical findings of the CFA and provided a transparent visual map of the MSQ structure [53]. This visual clarity is valuable for researchers, practitioners, and managers because it facilitates obtaining a deeper understanding of how specific items relate to each other and form meaningful clusters [54,55]. The successful use of this technique demonstrates that it is a novel and robust method for performing instrument validation in this specific cultural and provisional setting [35,52].

### 4.2. Practical Implications

Validating the twelve-item, three-factor MSQ-SF in the Romanian healthcare context provides actionable insights for organizational management, human resources, and policy-makers. Firstly, the clear differentiation of the Supervisory Relationship factor means that management must recognize the distinct importance of direct supervisors’ competence and conduct. Unlike intrinsic factors, this relationship is externally controllable and can be directly influenced by targeted leadership training programs focused on effective delegation and clear communication. For instance, human resources departments can use this validated instrument to benchmark supervisory performance and tailor interventions. Secondly, the confirmed importance of Autonomy Satisfaction and Task Enrichment suggests that simple financial rewards alone are insufficient to ensure job satisfaction. Hospitals and clinics can improve job satisfaction by redesigning roles to increase variety (MSQ3) and task control (MSQ2) and by linking performance to professional growth opportunities (MSQ14). This locally validated tool can be used to monitor the effectiveness of these structural and relational interventions in real time, allowing for evidence-based decision-making in organizational development [17,22]

### 4.3. Limitations

Despite its robust psychometric findings, this study has several limitations that constrain the generalizability and scope of the results.

First, the non-random sampling approach, confined to a single private healthcare institution in Romania, limits the generalizability of the findings to the broader Romanian workforce [15,16]. The positive reputation and specific context of this institution may give it unique characteristics that influence employee satisfaction in a different manner than the public sector or other private sector organizations.

Second, a significant limitation was the inability to perform a full Structural Equation Modeling (SEM) analysis with demographic predictors due to persistent issues with empirical non-identification and numerical instability. Despite extensive troubleshooting, the model could not provide reliable estimates of the structural paths from variables such as age and gender to the latent satisfaction factors. Key issues include a non-positive definite W matrix, which prevents the computation of robust standard errors, and the appearance of infinite factor covariances [56]. These problems likely stem from the inherently high correlation between the first two latent factors and the highly skewed, non-normal nature of ordinal (Likert-scale) data.

### 4.4. Further Works

Future research should extend the present findings to a broader and more representative sample of the Romanian healthcare sector, including multiple public and private institutions, to enhance the generalizability of the validated three-factor model. A larger and more diverse sample would also provide the statistical power necessary to address the empirical non-identification issues encountered in our SEM analysis [56].

The striking conceptual similarity between the Romanian factor structure and that of the Greek-Cypriot validation study suggests a compelling avenue for regional comparative research [9]. Future studies should investigate whether this factor structure is a geo-social phenomenon by exploring job satisfaction dimensions across the Balkans and South-Eastern European regions.

As science progresses into the digital and Artificial Intelligence era, these pencil-and-paper tools are evolving into digital instruments that will collect, analyze, and deliver results in a digital and automatic manner; this process has already been seen in different domains, such as preventive medicine [46], suggesting a clear direction for guiding future interest in this area.

## 5. Conclusions

The MSQ-SF is a widely used and highly regarded tool for assessing job satisfaction in various professional contexts. This study successfully validated a new context-specific factor structure in the Romanian organizational environment. By demonstrating that the original two-factor model of intrinsic and extrinsic satisfaction had an inadequate fit for the data, our findings align with the growing body of international literature underscoring the importance of local psychometric validation. We identified a robust and psychometrically sound three-factor model consisting of Task Enrichment, Autonomy Satisfaction, and Supervisory Relationships, which offers a more nuanced and culturally relevant framework for understanding job satisfaction in Romania. This new model provides researchers and organizational practitioners with a reliable and valid tool for measuring the key dimensions of employee satisfaction, enabling more effective interventions and human resource strategies.

The analysis of the construct validity provided strong support for the proposed three-factor model. Both EFA and CFA demonstrated an excellent fit to the data, as evidenced by the robust fit indices (CFI = 0.992, TLI = 0.989, RMSEA = 0.055, SRMR = 0.03). The Internal Consistency of the overall scale (Cronbach’s α = 0.933) and its individual factors was also high enough to provide a strong basis for reliability.

## Figures and Tables

**Table 1:** A summary of previous factor solutions found in the literature.

Author	Weiss (Original) [4]	Walkowiak et al., 2019 [11]	Martins, H., 2012 [10]	Huynh et al., 2024 [13]	Lakatamitou et al., 2020 [9]
Country	USA	Poland	Portugal	Vietnam	Cyprus
MSQ1	Intrinsic	Intrinsic	***	Specificity	***
MSQ2	Intrinsic	Intrinsic	Supervisor/Empowerment	Specificity	Supervisor/Autonomy
MSQ3	Intrinsic	Intrinsic	Task Enrichment	Specificity	Supervisor/Autonomy
MSQ4	Intrinsic	Intrinsic	Supervisor/Empowerment	Autonomy	Supervisor/Autonomy
MSQ5	Extrinsic	Extrinsic	Supervisor/Empowerment	Autonomy	***
MSQ6	Extrinsic	Extrinsic	Supervisor/Empowerment	***	***
MSQ7	Intrinsic	Intrinsic	***	***	Supervisor/Autonomy
MSQ8	Intrinsic	(Intrinsic)	***	***	Supervisor/Autonomy
MSQ9	Intrinsic	Intrinsic	***	Autonomy	Supervisor/Autonomy
MSQ10	Intrinsic	Intrinsic	Task Enrichment	Autonomy	Supervisor/Autonomy
MSQ11	Intrinsic	Intrinsic	Task Enrichment	Autonomy	Supervisor/Autonomy
MSQ12	Extrinsic	Extrinsic	***	***	***
MSQ13	Extrinsic	Extrinsic	***	***	Task Enrichment
MSQ14	Extrinsic	Intrinsic	***	Obligation	Task Enrichment
MSQ15	Intrinsic	Intrinsic/Extrinsic	Task Enrichment	Obligation	Task Enrichment
MSQ16	Intrinsic	Intrinsic	Task Enrichment	Obligation	Task Enrichment
MSQ17	General	Extrinsic	***	Obligation	Task Enrichment
MSQ18	General	(Extrinsic)	***	Autonomy	***
MSQ19	Extrinsic	Extrinsic	Task Enrichment	***	Task Enrichment
MSQ20	Intrinsic	Intrinsic	***	Autonomy	Task Enrichment
Number Items Discarded	0	0	10	6	5

***—Discarded items during the validation process.

**Table 2:** The general demographic and professional characteristics of the study population (*n* = 435).

				95% CI For Percentage	
Characteristic		Frequency (*N*)	Percentage (%)	Lower Bound	Upper Bound
Gender	Male	362	83.21%	79.74%	86.70%
	Female	58	13.33%	10.12%	16.55%
	No Answer	15	3.44%	1.71%	5.18%
Age	30 Years Old and Younger	94	21.60%	17.67%	25.53%
	31 to 40 Years Old	87	20.00%	16.21%	23.79%
	41 to 50 Years Old	87	20.00%	16.21%	23.79%
	51 Years Old and Above	100	22.98%	18.94%	27.04%
	No Answer	67	15.40%	12.03%	18.77%
	Mean ± Standard Deviation	40.81 ± 11.87			
	Median	41			
	Youngest Age	19			
	Oldest Age	68			
	25th Percentile	30			
	75th Percentile	51			
Education Level	Primary School	1	0.23%	0.00%	0.63%
	Middle School	10	2.29%	1.30%	0.33%
	College	110	25.29%	21.28%	29.30%
	Tertiary Education	294	67.58%	63.18%	72.00%
	No Answer	20	4.60%	3.26%	5.94%
Leading Role	Yes	26	5.97%	4.45%	7.51%
	No	400	91.95%	89.50%	94.40%
	No Answer	9	2.06%	1.13%	3.01%
Employment Status	Full-Time	369	84.82%	81.47%	88.19%
	Part-Time	48	11.03%	8.05%	14.01%
	No Answer	18	4.13%	2.87%	5.41%
Time Since Initial Employment in The Surveyed Institution [Months]	Employed for Less Than 3 Years	254	58.39%	53.72%	63.06%
	Employed for 3–5 Years	63	14.48%	11.20%	17.76%
	Employed for Over 5 Years	99	22.75%	18.72%	26.80%
	No Answer	19	4.36%	3.06%	5.68%
	Mean ± Standard Deviation [Months]	42.74 ± 38.53			
	Median [Months]	31			
	Newest Employed in Institution [Months]	1			
	Oldest Employed in Institution [Months]	168			
	25th Percentile	12			
	75th Percentile	60			
Time Since Employment in The Workforce [Months]	Mean ± Standard Deviation [Months]	85.78 ± 101.95			
	Median [Months]	44			
	Newest in Workforce [Months]	1			
	Oldest in Workforce [Months]	502			
	25th Percentile	19			
	75th Percentile	111			
	No Answer	22	5.32%		
Activity Domain	Daycare Fields	136	31.26%	26.85%	35.67%
	Surgical Fields	91	20.92%	17.05%	24.79%
	Clinical Fields	86	19.77%	16.02%	23.52%
	Customer Service	76	17.47%	13.92%	21.02%
	Technical and Administrative Fields	41	9.42%	6.65%	12.21%
	No Answer	5	1.14%	0.39%	1.91%
Professional Background	Healthcare Professional	296	68.04%	63.67%	72.43%
	Administration and Economics	43	9.88%	7.04%	12.74%
	Technical	27	6.20%	4.64%	7.78%
	Services	20	4.59%	3.26%	5.94%
	Military	3	0.69%	0.16%	1.22%
	Undergraduate	17	3.90%	2.67%	5.15%
	No Answer	29	6.66%	5.04%	8.30%
Current Job Title Category	Doctors and Pharmacists	44	10.11%	7.23%	12.99%
	Nurses	161	37.01%	32.37%	41.65%
	Nursing Aides	80	18.39%	14.74%	22.04%
	Support Staff	86	19.77%	16.02%	23.52%
	Technical Support	23	5.28%	3.86%	6.72%
	Administrative Staff	26	5.97%	4.45%	7.51%
	No Answer	15	3.44%	2.30%	4.60%

**Table 3:** Factor loadings after the Oblimin rotation.

	Factor 1	Factor 2	Factor 3	Uniqueness
MSQ13	0.864			0.336
MSQ19	0.847			0.292
MSQ15	0.822			0.233
MSQ16	0.806			0.324
MSQ20	0.735			0.328
MSQ14	0.697			0.376
MSQ3		0.952		0.209
MSQ9		0.631		0.485
MSQ4		0.605		0.312
MSQ2		0.602		0.443
MSQ6			1.02	0.005
MSQ5			0.695	0.233
Percentage of variances before rotation	59.3%	6.5%	4.4%	
Percentage of variances after rotation	35.9%	19.8%	14.5%	
Eigenvalues	7.41	0.929	0.835	

## Data Availability

The original contributions presented in this study are included in the article. Further inquiries can be directed to the corresponding author.

## Abbreviations

The following abbreviations are used in this manuscript:

MSQ	Minnesota Satisfaction Questionnaire
MSQ-SF	Minnesota Satisfaction Questionnaire–Short Form
JSS	Job Satisfaction Questionnaire
eNPS	Employee Net Promoter Score
CFA	Confirmatory Factor Analysis
EFA	Exploratory Factor Analysis
AVE	Average Variance Extracted
MCAR	Missing Completely at Random
KMO	Kaiser–Meyer–Olkin
CFI	Comparative Fit Index
TLI	Tucker–Lewins Index
NFI	Bentler–Bonett Normed Fit Index
RMSEA	Root Mean Square Error of Approximation
SRMR	Standardized Root Mean Square Residual
HTMT	Heterotrait–Monotrait Ratio
PNFI	Parsimonious Normed Fit Index
RFI	Relative Fit Index
IFI	Incremental Fit Index
RNI	Relative Noncentrality Index
GFI	Goodness-of-Fit Index
PDI	Power Distance

## Appendix A

**Table A1:** Item abbreviation and the Romanian translation from the original Minnesota Satisfaction Questionnaire–Short-Form.

Item Abbreviation	Question
MSQ1	*Posibilitatea de a avea tot timpul ceva de făcut* Being able to keep busy all the time
MSQ2	*Oportunitatea de a lucra singur, independent.* The chance to work alone on the job
MSQ3	*Oportunitatea de a face lucruri diferite din când în când.* The chance to do different things from time to time
MSQ4	*Oportunitatea de a fi recunoscut în comunitate.* The chance to be “somebody” in the community
MSQ5	*Modul în care șeful meu își tratează angajații*. The way my boss handles his/her workers
MSQ6	*Competența șefului meu direct în luarea deciziilor.* The competence of my supervisor in making decisions
MSQ7	*Posibilitatea de a face lucruri care nu contravin principiilor mele morale*. Being able to do things that do not go against my conscience
MSQ8	*Modul în care locul meu de muncă oferă stabilitate.* The way my job provides for steady employment
MSQ9	*Oportunitatea de a face lucruri pentru alți oameni.* The chance to do things for other people
MSQ10	*Oportunitatea de a da indicații altora.* The chance to tell people what to do
MSQ11	*Oportunitatea de a-mi valorifica abilitățile.* The chance to do something that makes use of my abilities
MSQ12	*Modul în care politicile instituției sunt puse în practică.* The way company policies are put into practice
MSQ13	*Salariul în raport cu volumul de muncă.* My pay and the amount of work I do
MSQ14	*Oportunitățile de promovare în acest loc de muncă.* The chances for advancement on this job
MSQ15	*Libertatea de a-mi folosi propria judecată.* The freedom to use my own judgment
MSQ16	*Posibilitatea de a încerca propriile metode pentru îndeplinirea sarcinilor de serviciu.* The chance to try my own methods of doing the job
MSQ17	*Condițiile de muncă.* The working conditions
MSQ18	*Modul în care colegii mei se înțeleg între ei.* The way my co-workers get along with each other
MSQ19	*Aprecierea primită pentru o muncă bine făcută.* The praise I get for doing a good job
MSQ20	*Sentimentul de împlinire pe care mi-l oferă locul de muncă.* The feeling of accomplishment I get from the job
MSQ21 (SATGEN)	*În general, cât de mulțumit sunteți la acest loc de muncă?* Overall, how satisfied are you with this job?
Very Satisfied	*Foarte mulțumit*
Satisfied	*Mulțumit*
Neither Satisfied nor Dissatisfied	*Nici mulțumit, nici nemulțumit*
Dissatisfied	*Nemulțumit*
Very Dissatisfied	*Foarte nemulțumit*
